# Conductometric Chemosensor for Saccharides Based on Thin Films of Poly(3-Thienylboronic) Acid: Measurements of Transversal Resistance

**DOI:** 10.3390/bios15100679

**Published:** 2025-10-09

**Authors:** Berfinsu Kaya, Yulia Efremenko, Vladimir M. Mirsky

**Affiliations:** Department of Nanobiotechnology, Institute of Biotechnology, Brandenburg University of Technology Cottbus-Senftenberg, 01968 Senftenberg, Germany; berfinsu.kaya@b-tu.de (B.K.); iullia.efremenko@b-tu.de (Y.E.)

**Keywords:** chemical sensor, saccharide sensor, 3-thienylboronic acid, conductometric sensor, electrochemical impedance spectroscopy

## Abstract

Poly(3-thienylboronic acid) (PThBA) has recently been suggested as a conducting polymer with affinity for saccharides. In this study, thin films of this compound were deposited onto gold electrodes. The system obtained was studied as a possible chemical sensor. The measurements were performed by impedance spectroscopy using potassium ferro/ferricyanide as a redox mediator. The thickness of the polymer and the deposition of the adhesive sublayer were optimized to achieve a compromise between the blocking of defects in the polymer layer and the unnecessary increase in the internal resistance of this conductometric sensor. A comparative study of the influence of fructose, glucose, and sorbitol on transversal polymer resistance was conducted. The binding constants for these saccharides were extracted from the concentration dependencies of sensor conductance. Among them, sorbitol showed the highest affinity with a binding constant up to ~15,000 L·mol^−1^, followed by fructose (~8700 L·mol^−1^) and glucose (~4500 L·mol^−1^). In order to exclude the contribution of the analyte tautomers on the obtained binding constants, measurements of ethylene glycol were also performed. The effects of pH and the redox state of PThBA on its affinity properties were studied, revealing higher affinities at alkaline pH and in oxidized state of the chemosensitive polymer. The developed system has the capacity to be applied in chemical sensors and virtual sensor arrays with electrical affinity control.

## 1. Introduction

Saccharides, more commonly referred to as carbohydrates, are the principal energy sources and structural elements in living organisms. Monosaccharides such as glucose are fundamental to cellular metabolism, supplying essential energy for several physiological functions [1]. The precise detection and quantification of saccharides is imperative in a variety of applications, including biomedical diagnostics, food safety, and environmental monitoring [2]. For instance, glucose monitoring is critical for the management of diabetes [3], while the detection of ethylene glycol is necessary due to the toxicity of its breakdown products and potential for poisoning [4,5].

Traditional methods for saccharide analysis, including enzyme-based sensors, electrochemical techniques, and spectroscopic methods, face several limitations. However, enzyme-based sensors frequently exhibit limitations in terms of their lifespan and stability when exposed to extreme temperatures, pH variations, or in the presence of detergents and solvents. This can pose significant challenges during the sterilization process [6]. Spectroscopic methods [7,8] may lack the necessary sensitivity for detecting low concentrations and can be affected by sample turbidity or color, limiting their applicability in certain scenarios.

Non-enzymatic electrochemical methods have been explored for glucose detection to overcome these challenges. These approaches focus on the direct oxidation of glucose on electrode surfaces without using biological enzymes, thereby enhancing sensor stability [9,10,11,12,13]. Electrocatalytic approaches for saccharide detection have been the subject of extensive investigation. The utilization of alternative electrocatalytic materials, including gold nanoparticles, has been proposed as a potential solution. However, these methods frequently encounter issues such as limited selectivity and electrode fouling due to non-specific adsorption of interfering species. Consequently, there is a growing interest in the development of affinity sensors that utilize specific interactions between glucose and synthetic receptors or materials, with a view to improving selectivity and reducing electrode fouling. Boronic acid derivatives have been extensively utilized for saccharide detection, as a consequence of their capacity to bind reversibly to diol-containing compounds through covalent interactions [14,15,16,17]. The interaction of boronic acids with saccharides leads to the formation of cyclic boronate esters, which provide selective recognition and quantification of target analytes in aqueous solutions.

Boronic acids are defined as organic molecules consisting of a boron atom covalently bound to an alkyl or aryl group and two hydroxyl groups, resulting in the typical structure of R-B(OH)_2_. Boronic acids are distinguished by their ability to form reversible covalent bonds with diol-containing molecules, such as saccharides and catechol. This interaction entails the synthesis of cyclic boronate esters via the reaction between boronic acid and the cis-diol groups of the target molecules [18]. The binding affinity and specificity of these interactions are affected by pH and the properties of substituents. Research indicates that electron-withdrawing groups on the aromatic ring of phenylboronic acids can augment their binding affinity for diols by elevating the acidity of the boronic acid, thereby promoting ester formation [15,19]. 

The distinctive diol-binding characteristics of boronic acids have been utilized in the creation of electrochemical sensors for the identification of saccharides and other diol-containing biomolecules [20,21,22]. In these sensors, boronic acids are immobilized on electrode surfaces, facilitating the selective identification of target analytes via boronate ester formation. This binding event has the potential to modify the electrochemical characteristics of the electrode interface. 

However, despite the potential applications of boronic acid-based electrochemical sensors, certain limitations were identified. A significant problem is too low binding affinity of some boronic acids for certain diol-containing molecules. This phenomenon may compromise the performance of sensors by reducing their sensitivity and selectivity [20,21]. The binding interactions are usually pH-dependent [19] with optimal binding occurring in alkaline conditions. This limitation restricts the applicability in physiological conditions. The present research endeavors to synthesize the boronic acid derivative [23,24] that exhibits enhanced binding affinity and reduced pH influence.

A range of derivatives of boronic acid [25] and polymers containing boronic acid moiety [26,27] were suggested and studied. However, it should be noted that most of these polymers are not electrically conducting or electrochemically active, thus hindering their effective applications in conductometric transducers or electrochemical chemotransistors. This hindrance also precludes the application of these compounds in smart chemical sensors, which offer fast sensor recovery [28], electrical affinity control [29] or virtual sensor arrays [30]. It was the motivation to develop new receptor material based on conducting polymers with boronic acid moieties—poly-3-thienylboronic acid (PThBA) [17,20].

PThBA is the polythiophene derivative that has been modified with a chemosensitive boronic acid group to provide a binding affinity for saccharides and other diol-containing substances. Electrochemical polymerization of 3-thienylboronic acid (ThBA) to synthesize PThBA from its solutions, which are often used for electrochemical synthesis of other polymers, was unsuccessful due to the limited solubility and reactivity of ThBA. The problem was resolved by electropolymerization in the solution based on the combination of boron trifluoride diethyl etherate (BFEE) and acetonitrile (ACN) with the addition of 2,6-di-*tert*-butylpyridine (DTBP): high-quality polymer layers were obtained [31]. This approach, which entailed the deposition of an adhesive sublayer to form thin polymer films on the electrode surface, was further developed in [32] for the purpose of producing free-standing films with advanced thermoelectric properties. PThBA has been successfully fabricated in different structural forms, including thin polymer layers [17,31], free-standing polymeric films [17,32], or nanoparticles [33]. This structural versatility enhances its potential utility across a range of sensing applications. 

PThBA provides notable benefits, including its compatibility with optical and conductometric signal transduction methods and the ability for electrochemical modulation of its affinity [17,33], which enables the way for extension of the detection range, fast sensor recovery, or formation of virtual sensor arrays. PThBA-based sensors show significant sensitivity within the millimolar concentration range [17], a property that renders them especially pertinent for biomedical applications. In contrast to phenylboronic acid-based sensors that predominantly bind fructose [34,35], PThBA sensors exhibit affinity to many saccharides, including glucose [17], thus broadening their possible applications in clinical diagnostics, biotechnology, and food industry. 

Prior research on the PThBA-based conductometric sensor was focused on the measurement of lateral (in-plane) resistance. That approach has important advantages, such as an internal integrity control via simultaneous two- and four-point measurements [36,37] as well as a possibility to measure electrical resistance without the addition of a redox mediator. However, there is a fundamental question regarding the feasibility of signal transduction based on the transversal resistance of PThBA, measured perpendicularly to the electrode surface. Such a configuration was previously reported for saccharide receptors immobilized on the gold electrodes based on various derivatives of phenylboronic acid [38,39]. In this study, we investigate PThBA as a sensing layer using transducers that operate through the measurement of transversal conductance (impedance).

## 2. Materials and Methods

### 2.1. Materials 

3-thienyboronic acid (ThBA), boron trifluoride diethyl etherate (BFEE), 2,6-di-*tert*-butylpyridine (DTBP), thiophenol, D-sorbitol, D-fructose, D-(+)-glucose, ethylene glycol, as well as potassium hexacyanoferrate III and potassium hexacyanoferrate II trihydrate were obtained from Sigma-Aldrich (Darmstadt, Germany). Ethanol was purchased from Th. Geyer (Renningen, Germany), acetonitrile (ACN), sodium hydrogen phosphate, sodium dihydrogen phosphate, and sodium chloride from ROTH (Karlsruhe, Germany). 

The pH of the buffer solutions was adjusted with 1 M solutions of NaOH or HCl from Roth (Karlsruhe, Germany). All aqueous solutions were prepared with deionized water and further purified using the ELGA-Classic system (Hermsdorf, Germany). Purchased chemicals were used as they were received. 

### 2.2. Instrumentation and Procedures

Before electropolymerization, gold electrodes were cleaned using piranha solution: a mixture of 30% H_2_O_2_ and concentrated H_2_SO_4_ in a 1:3 (*v*/*v*) ratio. Caution: this mixture reacts violently with most organic materials and must be handled with extreme care. The cleaned electrode was rinsed thoroughly with deionized water and dried under nitrogen flow before modification. 

To improve polymer adhesion during electrochemical polymerization, the electrode surface was pre-coated with a self-assembled monolayer of thiophenol by immersing the electrode in 10 mM thiophenol in ethanol for 30 min. The electrode was then rinsed with ethanol and dried. 

The polymerization of ThBA was carried out using a standard three-electrode configuration. The working electrode was immersed in a 0.05 M solution of ThBA dissolved in the mixture of 90% BFEE and 10% ACN (*v*/*v*), containing 0.05 M DTBP as a proton scavenger. Electropolymerization was performed via potential cycling from −0.2 V to +1.8 V versus an Ag/AgCl reference electrode Metrohm (Herisau, Switzerland) filled with 2 M LiCl in ethanol, with the same electrolyte used in the salt bridge. A carbon counter electrode was used to complete the three-electrode setup. 

The gold wire of 99,999% purity from ABCR (Karlsruhe, Germany) used for the deposition of the polymer had a length of 1.1 cm and a diameter of 0.5 mm. Electropolymerization was performed using the Autolab PGSTAT-12 General Purpose Electrochemical System (Champaign, IL, USA) with Nova 2.1.4 software to control the device and to analyze the impedance spectra. The Ag/AgCl reference electrode with a double salt bridge (saturated KCl) was used for the characterization of the sensor. The measurements were performed at room temperature (~24 °C) in the electrolyte containing 2 mM potassium ferro/ferricyanide (Fe(CN)_6_^3−^/^4−^), 1 M KCl, and 100 mM buffer solution. The type of buffer utilized and the pH value are specified in the descriptions of specific experiments. In oxygen-free impedance experiments, before each measurement, argon 99.9999% BIP (Calau, Germany) was bubbled for 20–40 min through all solutions used in this experiment. All measurements were conducted at ambient temperature. Potential values are presented relative to the reference electrode used.

Scanning electron microscopy (SEM) was carried out using ZEISS EVO MA 15 (Oberkochen, Germany) operated at an accelerating voltage of 15.00 kV with a working distance of 11.5 mm. All images were obtained using a secondary electron detector to provide detailed topographical contrast. Samples were fixed on conductive carbon tape and analyzed without additional coating, as the polymer layer provided sufficient conductance.

## 3. Results and Discussion

### 3.1. Electrochemical Deposition of Thin Films of PThBA 

**Figure 1:**
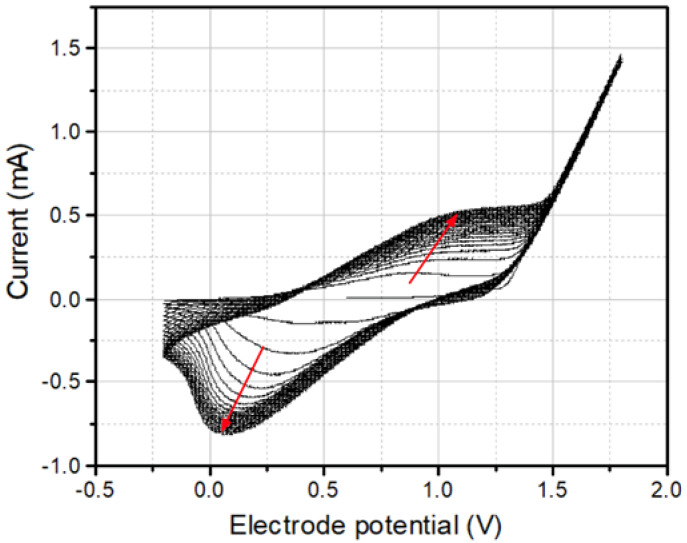
Cyclic voltammetry of PThBA electropolymerization on the gold electrode. Deposition was performed over 20 consecutive cycles, within a potential window of −0.2 V to +1.8 V (vs. Ag/AgCl) at a scan rate of 0.1 V/s. The arrows indicate peak evolution during the deposition. Electrolyte: 50 mM 3-thienylboronic acid dissolved in the 9:1 (*v*/*v*) mixture of boron trifluoride diethyl etherate and acetonitrile with addition of 2,6-di-*tert*-butylpyridine.

The deposition of PThBA onto gold electrodes was performed using cyclic voltammetry-based electropolymerization, a well-established technique for forming thin polymer films [31]. The cyclic voltammogram (CV) obtained during the electropolymerization process (Figure 1 and Figure S1) exhibits a continuous increase in peak current with each cycle, thus validating the incremental deposition of a conducting polymer layer on the electrode surface. The polymerization was performed in the solution of ThBA in the mixture of BFEE and ACN containing DTBP as a proton scavenger within a potential range from −0.2 V to +1.8 V (vs. Ag/AgCl). 

Preliminary experiments have demonstrated that PThBA adhesion to the gold surface is relatively poor. A monomolecular film of thiophenol was applied before electrochemical polymerization to obtain stable polymer coating. This approach was suggested earlier for the deposition of polyaniline [35] and was then applied for the deposition of PThBA for chemical sensors with lateral conductometric transducing [17,31]. The cyclic voltammograms derived from ThBA polymerization on thiophenol-coated gold electrodes are close to those obtained for the electropolymerization on bare gold surfaces (Figure S1), but with some lower values of electrical current.

**Figure 2:**
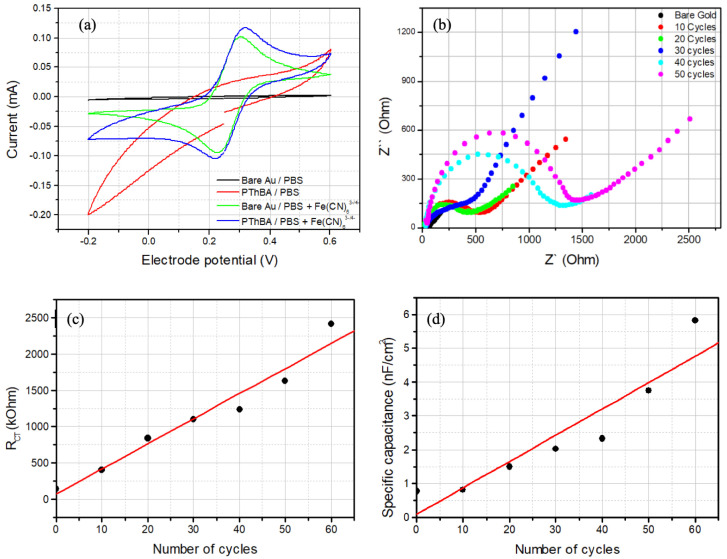
Cyclic voltammograms recorded at 0.1 V/s in phosphate buffer (PBS, pH 7.4), demonstrating the effect of PThBA coating and the presence of ferro/ferricyanide in the electrolyte on the electrochemical response of gold electrodes (**a**), examples of impedance spectra presented as Nyquist plots for different number of cycles (**b**), as well as the film resistance (**c**) and capacitance (**d**) values extracted from the impedance spectra. The impedance spectroscopy was performed in the frequency range from 0.1 Hz to 1 MHz at the electrode potential of +0.2 V. The error bars indicate the standard deviation in the fitting of the corresponding impedance spectrum. Electrolyte: 2 mM potassium ferro/ferricyanide (Fe(CN)_6_^3−^/^4−^), 1 M KCl.

The application of cyclic voltammetry to PThBA-coated electrodes in an aqueous electrolyte did not result in the observation of oxidation or reduction peaks (Figure 2a, red curve). This phenomenon is analogous to the behavior exhibited by polythiophene [40]. In the presence of ferro/ferricyanide, the usual redox activity was observed (Figure 2a). However, PThBA deposition resulted in a decrease in the oxidation and reduction peaks and an increase in the gap between these peaks (Figure 2a, blue and green curves). This finding suggests that the coating enhances the resistance of the electrochemical reaction. Despite the preservation of electrical conductance, the polymer establishes a barrier that hinders electron transfer and Ohmic drops within the polymer layer.

Impedance spectroscopy of the PThBA-coated gold electrodes was subsequently conducted (Figure 2b). At high frequencies, the Randles circuit can describe the spectra, which includes a serially connected resistance (e.g., the resistance of the electrolyte, reference electrode, etc.) with a parallel connected resistance (e.g., the charge transfer resistance, RCT) and capacitance. Further, this RCT value is regarded as the film resistance. At lower frequencies, a characteristic tail indicating the Warburg impedance is observed. The values of the film resistance and capacitance were extracted from the high-frequency part of the impedance spectra (Figure 2c,d).

As illustrated in Figure 2c, there is a direct correlation between the film resistance and the number of potential cycles during electrochemical polymerization. A clear increase in the resistance with increasing cycle number is observed. This finding aligns with the CV data (Figure 2a), suggesting that the polymer layer forms a passive barrier to charge transfer. An increase in the number of polymerization cycles results in an increase in the thickness of the PThBA layer and in the filling of defects in the polymer film. Despite the presence of electrical conductance and electrochemical activity, the layer resistance is sufficiently high to impede electron transfer.

The measured value of the capacitance may encompass geometric capacitance and Faradaic pseudo-capacitance. The geometric capacitance also includes the serially connected capacitance of the ionic double layer. For a film derived from a polymer that is not electrochemically active, an increase in the polymer layer would result in a decrease in the film capacitance. In most cases, the double-layer capacitance is much larger than the coating capacitance. In this case, the total capacitance is approximately reciprocally proportional to the film thickness. Conversely, Faradaic pseudo-capacitance is directly proportional to the total amount of electrochemically active compound on the electrode. Consequently, an increase in electrode capacitance with increasing polymerization cycles can be anticipated. In the experiment, a linear (correlation coefficient 0.99) increase in the capacitance with the number of polymerization cycles was observed (Figure 2d); each polymerization cycle added ~14 µF/cm^2^ to the electrode capacitance. The specific capacitance was found to be in the range of ~mF/cm^2^, which is at least an order of magnitude higher than the capacitance of the ionic double layer and a few orders of magnitude higher than the geometrical capacitance of films with a thickness of hundreds of nm. Therefore, the measured capacitance corresponds to the Faradaic pseudo-capacitance, and its monotonous increase characterizes an increase in the amount of redox-active compound with each voltammetric cycle during electrochemical polymerization.

**Figure 3:**
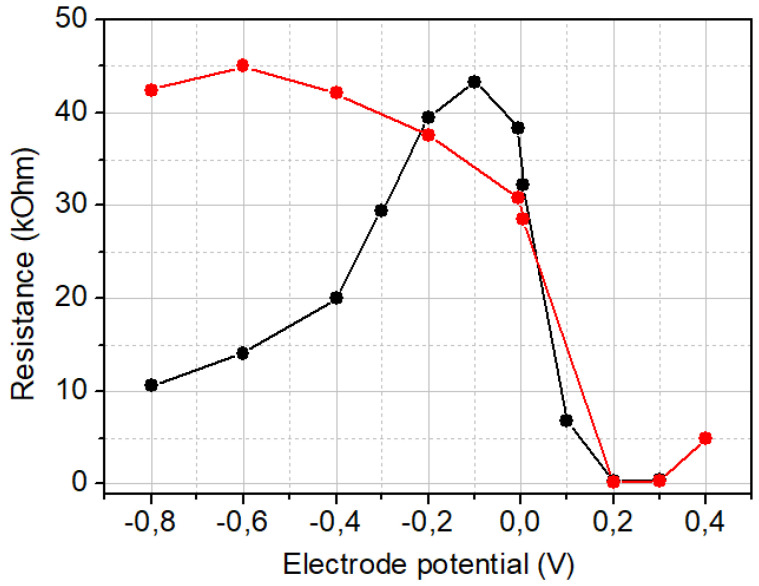
Influence of the electrode potential on the resistance of PThBA film deposited during 20 voltammetric cycles. The resistance values were extracted from impedance spectra measured under aerobic (red circles) and anoxic (black circles) conditions at pH 7.4.

PThBA belongs to the category of conducting electrochemically active polymers [17,31]. Consequently, the redox state of the polymer deposited on the electrode can be regulated by the electrode potential. Considering the chemosensitive properties of this polymer and its potential application in smart chemosensitive devices with electrical affinity control (e.g., virtual sensor arrays [30] or chemical sensors with accelerated sensor recovery [28]), we analyzed PThBA film resistance measurements at different electrode potentials. The results are presented in Figure 3. Notably, the results depend on the presence of oxygen. Under aerobic conditions (Figure 3, red circles), two polymer states with different conductivities were observed: a low-conducting state at an electrode potential below +0.1 V and a high-conducting state at an electrode potential above +0.1 V. Under anoxic conditions (Figure 3, black circles), three conductance ranges can be observed, which may correspond to three redox states of PThBA under these conditions: a conducting state under reduced conditions at an electrode potential below −0.3 V, a low-conducting state between −0.3 V and +0.1 V, and a highly conducting, oxidized state at an electrode potential above +0.1 V. The presence of oxygen results in the conversion of the conducting stage at cathodic potentials into a low-conducting stage. However, this conversion does not influence the redox equilibrium at anodic potentials. 

**Figure 4:**
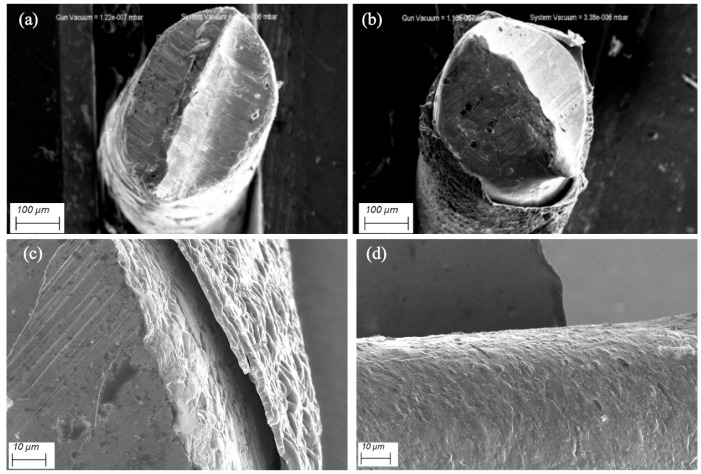
SEM images of the gold electrode cut with a wire cutter before coating (**a**) and after deposition of PThBA during 20 potentiometric cycles (**b**–**d**).

To assess the morphological evolution of the electrode surface due to polymer deposition, scanning electron microscopy (SEM) was performed (Figure 4). Cross-sectional SEM images obtained after the electrode was cut by the cutter pillar demonstrate a polymer layer of ~µm thickness (Figure 4b,c). The polymer surface is relatively rough (Figure 4d). The observed polymer detachment from the gold surface (Figure 4c) may be caused by sample preparation for SEM. 

Finally, the study based on cyclic voltammetry, impedance spectroscopy, and SEM indicates the formation of an electroactive polymer layer on the electrode surface. Although the PThBA possesses intrinsic electrical conductance, an increase in the layer thickness leads to an increase in the resistance of the polymer layer.

### 3.2. Effect of Fructose on the Resistance of PThBA Films in Aerobic and Anoxic Conditions

**Figure 5:**
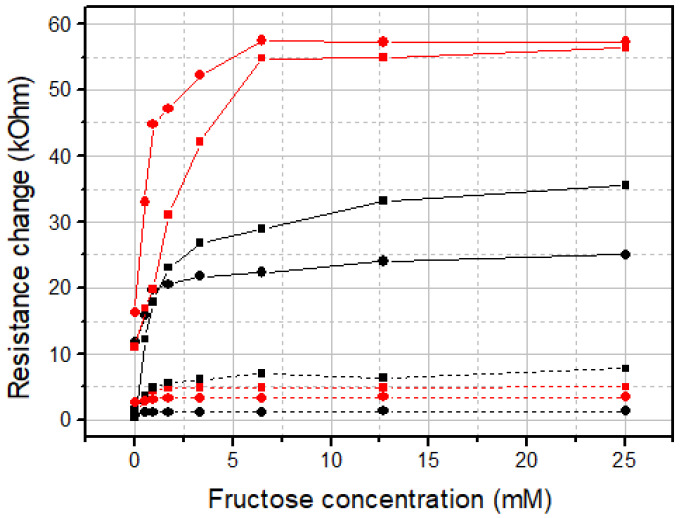
The effect of fructose on the resistance of the PThBA layer deposited within 20 (squares) and 50 (circles) voltammetric cycles in aerobic (black symbols connected by continuous lines) and anoxic (red symbols connected by continuous lines) conditions. The dotted lines correspond to the control measurements performed with the same electrolyte but without fructose. pH 7.4. Electrode potential: +0.2 V.

Resistance measurements of PThBA films under aerobic conditions demonstrated a permanent increase in this value for at least several hours. This drift complicates the quantitative measurements of an influence of saccharides on the polymer resistance. One can observe an increase in the resistance after additions of fructose with a saturation trend at concentrations above 5 mM (Figure 5). A more comprehensive illustration of the influence of various fructose concentrations on the electrical resistance and impedance spectra of PThBA-coated gold electrodes is shown in Figures S3 and S4. In anoxic conditions achieved by argon bubbling through the solutions, the resistance drift was smaller (Figure 5); therefore, all subsequent measurements were performed in anoxic conditions.

### 3.3. Influence of pH of the Electrolyte and Redox State of the Sensing Polymer on the Affinity Towards Various Saccharides

Boronic acid-functionalized molecules interact with cis-diols of the saccharides through reversible covalent binding, forming boronated esters. The stability of these esters depends on the pH of the environment. At alkaline pH, deprotonation of the boronic acid enhances its nucleophilicity, facilitating stronger interactions with diol-containing saccharides. This pH-dependent behavior is well-documented in the literature [19,20,41,42,43]; however, a deviation from this behavior was observed for the sensors based on the lateral resistance of PThBA [17]. Additionally, in the case of redox-active receptors, the affinity depends on the redox state of these compounds. This was the reason to study the influence of pH and electrode potential on the affinity, and also for the current sensor configuration. The selection of the electrode potentials for such measurements was based on the conductance dependence at anoxic conditions (Figure 3, black circles) so that each of the three conductance states was characterized: −0.5 V, −0.1 V, and +0.2 V. 

**Figure 6:**
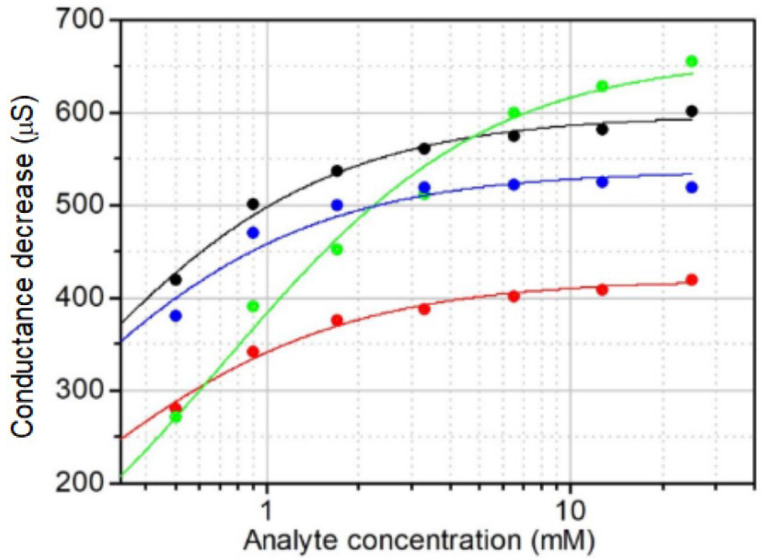
Fitting of concentration dependencies of conductance changes by Langmuir adsorption isotherms. Measurements were taken at a pH of 8.5 with an electrode potential of +0.2 V for sorbitol (black circles), glucose (red circles), ethylene glycol (green circles), and fructose (blue circles). The continuous lines show the fitting by the Langmuir adsorption isotherm. Figure S2 shows the fitting of the corresponding dependencies at pH 7.4 and 8.0 and electrode potentials of −0.5 V and −0.1 V.

The dependences of conductance changes on the analyte concentration obey the Langmuir adsorption isotherm (Figure 6 and Figure S2). The same dependence was obtained for the lateral resistance of PThBA in [17] and was explained by a simple electrical model of parallel resistors blocked by the saccharide binding.

**Figure 7:**
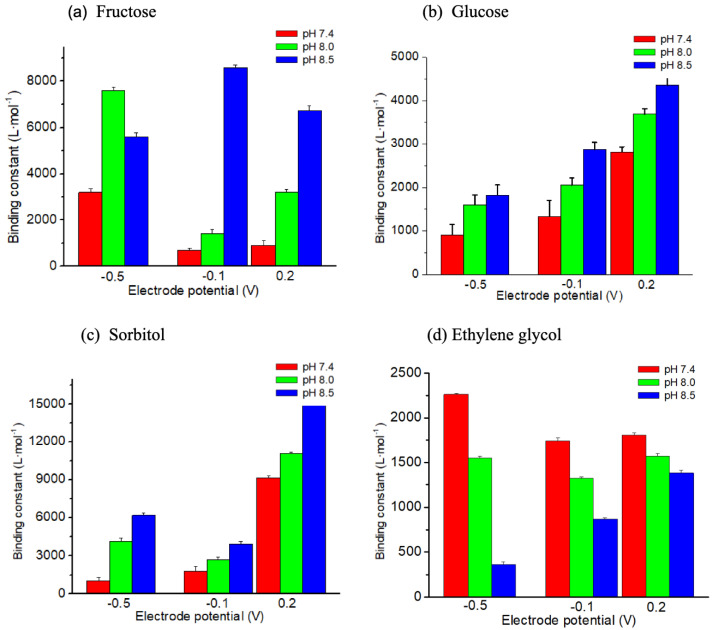
Influence of pH of the electrolyte and redox state of the sensing polymer on the affinity towards various saccharides and ethylene glycol.

Binding constants were calculated based on the fitting of experimental data by the Langmuir adsorption isotherm. These results are presented in Figure 7 and Figure S5.

The classical dependence of the affinity of the boronic acid moiety on pH, with affinity increase at alkaline pH, was observed only for saccharides. It was found for each redox state of the compound, except the affinity of fructose to PThBA in the reduced state of this polymer (Figure 7a). However, for ethylene glycol, the dependence was opposite: binding affinity decreased at alkaline pH. This suggests that the pH dependence mechanism may be more complex than merely influencing the stability of the boronic ester. All of the compounds studied, except ethylene glycol, can form multiple isomers, and not all of these forms can effectively react with the boronic acid moiety. The pH effect may also influence the equilibrium distribution of these forms of saccharides.

Sorbitol at pH 8.5 demonstrated the highest affinity (~15,000 L·mol^−1^). This value is higher than that observed for the same receptor in monomeric form (an interpolation of data from [19] gives a value of ~2000 L·mol^−1^) and for the polymeric form when lateral resistance was measured (an interpolation of data from [12] for pH 8.5 provides a value of ~4000 L·mol^−1^). Sorbitol affinity to PThBA depends strongly on pH and applied voltage (Figure 7c). At +0.2 V, a significant increase in binding is observed, with a value exceeding 16,000 L·mol^−1^ at pH 8.5. This is the highest value among all saccharides tested. At pH 7.4, binding remains relatively low at −0.5 V and −0.1 V but sharply increases at +0.2 V. This reveals that PThBA in the oxidized state has a higher affinity for sorbitol.

The highest binding constant for fructose, which is ~8700 L·mol^−1^, was observed at the middle value of the redox potential of PThBA at pH 8.5. This is also some higher than the binding constant for the monomeric and polymeric forms of this polymer—an interpolation of data from [24] and [17] gives the values of ~1700 L·mol^−1^ and ~3000 L·mol^−1^, respectively. At pH 8.5, a sharp increase in binding constant is observed, especially at −0.1 V. This may indicate that the alkaline conditions combined with the middle redox state of the receptor optimize the boronate ester formation with fructose. Interestingly, in more oxidizing conditions (+0.2 V), the affinity slightly decreases at pH 8.5. At pH 7.4, the binding remains consistently lower across all potentials, highlighting the known pH dependence of boronic acid-diol binding equilibria. The observed concentration dependence with consideration of signal drift (Figure 5) gives the value of the detection limit for this analyte ~0.1 mM.

The highest value of the binding constant for glucose was ~4500 L·mol^−1^, which was measured at alkaline pH and oxidic redox state of the receptor. The binding behavior of glucose shows a progressive pH- and potential-dependent increase (Figure 7b). At all pH values, increasing the potential from −0.5 V to +0.2 V correlates with a steady rise in binding constants. For the monomeric form of this receptor, the affinity to glucose was not measurable [24], and for the lateral resistance of PThBA, the affinity of ~2500 L·mol^−1^ was obtained at similar conditions (interpolation of data from [17] for pH 8.5). The comparison shows that the acquired data are close to the values obtained for the lateral resistance of PThBA. Some differences can be caused by a less direct approach to measuring resistance in the current work, which includes the impedance spectroscopy and subsequent analysis of the reaction resistance for oxidation/reduction of ferro/ferricyanide.

The data indicate that switching the redox state of PThBA to a more oxidative state (more anodic potential) by applying electrode potential increases the affinity of this chemosensitive material towards glucose at all studied pH levels. The effect on the affinity to the other materials studied was more complex and pH-dependent. These data allow us to consider implementing these effects in a virtual sensor array [30].

The ability to adjust affinity by changing pH or electrode potential can be used to accelerate sensor recovery [28], to extend the concentration range [17] or to improve the selectivity. The most advanced approach to improve the selectivity would involve recognizing the pH-potential response patterns. However, in binary mixtures a reasonable selectivity can be achieved just by appropriately selecting the measurement conditions. See Table 1 for some examples. Selectivity coefficients for the analytes in the presence of interfering compounds were calculated as the ratios of the corresponding binding constants. The data show that, except for glucose in the presence of sorbitol, it is possible to achieve prevailing sensitivity to the analyte in all considered cases. 

It should be noted that affinity is the intrinsic property of the material, which is independent of the method of investigation. At the same time, the affinity values reported here and in all other work on sensor applications are only the appeared values which are based on the data interpretation. In this study, as well as in [17], it is based on the postulate of validation of Langmuir adsorption isotherms. However, the detection method also contributes to the results obtained. The measurements of transversal resistance applied in [38,39] as well as in this investigation, have a relatively complex nature, being affected by the electrochemistry of ferro/ferricyanide. Despite the intensive study and broad applications of t ferro/ferricyanide redox system in various fields of electrochemistry, analytical chemistry, and sensor science, some aspects of the electrochemical behavior of this system are not clear so far. For example, there is still no complete explanation of the electrocatalytic activity of cations [44,45] or immobilized single-stranded DNA [46]. The electrochemistry of ferro/ferricyanide on PThBA coated electrodes is unknown. The absence of these fundamental data complicates a more exact analysis of the behavior of the PThBA based impedimetric sensor, in which ferro/ferricyanide is used for electrical coupling of charge transport. 

Previously, we studied the chemosensitive properties of this polymer by measuring the lateral polymer resistance under conditions that exclude any significant contribution from contact resistance. In that case, the measured values corresponded to the polymer resistance. The system studied in this work is more traditional, though more complex: a redox mediator is necessary to couple the conductance of the polymer and the electrolyte. Defects in the polymer layer may contribute to the measured resistance. Moreover, as shown in [33], saccharide binding leads to PThBA condensation. Thus, it is also conceivable that saccharides have an influence on the electrical current through defects. Therefore, the observed effects may include the influence of analyte binding on the polymer intrinsic conductance but also the changes in conductance through polymer defects due to the influence of the analyte on polymer volume.

The present study focuses on the evaluation of chemosensitive properties of PThBA applied in transversal measurement configuration in comparison with previously reported lateral configuration. The system for transversal resistance measurements does not require a fabrication of microstructured electrodes on a conducting support, the measurements can be performed by standard systems for impedance spectroscopy. For routine applications the measurement system can be simplified using AC measurements at single frequency; such a system in combination with flow cell will be more appropriate for kinetic measurements including analysis of the sensor response time and evaluation of kinetic constants of analyte binding and dissociation. In the same time, lateral resistance measurements [17] exploit the direct electron transfer between metallic electrode and chemosensitive conducting polymer, therefore does not require a presence of redox mediator and are less sensitive to possible analyte influence on the morphology of the receptor film.

## 4. Conclusions

The results confirm that the redox state of the PThBA films significantly affects their affinity for saccharides, especially fructose, glucose, and sorbitol. Redox switching via an applied electrode potential enables modulation of the binding behavior, with increased affinities observed at intermediate or oxidizing potentials. This study focused on analytical aspects and did not evaluate the exact mechanism of the observed effects.

Quantitative binding data extracted via Langmuir isotherm fitting revealed high binding constants for sorbitol (~15,000 L·mol^−1^), fructose (~8700 L·mol^−1^), and glucose (~4500 L·mol^−1^), particularly at alkaline pH and oxidized redox states of the chemosensitive polymer.

The present study confirms that in transversal configuration PThBA can also act as a chemosensitive material with electrical- and pH-tunable affinity. In comparison with monomeric form, the polymeric form exhibited considerably augmented binding constants, particularly for sorbitol and fructose. The influence of the pH and electrode potential on affinity properties can be used to improve the measurement selectivity, suggesting its potential application in complex sample analysis or multi-target sensing. 

Future directions may include miniaturizing this system, integrating it with microfluidic platforms, and adapting it for continuous glucose monitoring. The observed electrical modulation of PThBA’s affinity properties opens possibilities for selective extraction, affinity chromatography, and drug delivery systems where reversible boronate ester formation can be controlled electrically. Finally, this study underscores the significance of PThBA as a novel, highly versatile chemosensitive material with broad applications in analytical and biomedical fields.

## Figures and Tables

**Table 1:** Binary selectivity coefficients calculated from affinity constants.

Analyte	Interfering Compound	S_A/B_	pH	Electrode Potential, V
Fructose	Glucose	4.7	8.0	−0.5
	Sorbitol	3.2	7.4	−0.5
	Ethylene glycol	15	8.5	−0.5
Glucose	Fructose	3.1	7.4	0.2
	Sorbitol	0.89	7.4	−0.5
	Ethylene glycol	5.0	8.5	−0.5
Sorbitol	Fructose	10	7.4	0.2
	Glucose	3.4	8.5	0.2
	Ethylene glycol	17	8.5	−0.5
Ethylene glycol	Fructose	2.4	7.4	−0.1
	Glucose	2.5	7.4	−0.5
	Sorbitol	2.2	7.4	−0.5

## Data Availability

The original contributions presented in the study are included in the article or Supplementary Materials, further inquiries can be directed to the corresponding author.

## Supplementary Materials

The following supporting information can be downloaded at: https://www.mdpi.com/article/10.3390/bios15100679/s1, Figure S1. Cyclic voltammetry of electrochemical polymerization of PThBA on the bare gold electrode (red) and on the thiophenol-coated gold electrode (black). Deposition was performed over 20 consecutive cycles, within a potential window of −0.2 V to +1.8 V (vs. Ag/AgCl) at the scan rate of 0.1 V/s. Electrolyte: 50 mM ThBA dissolved in the mixture of 90% BFEE and 10% ACN (*v*/*v*); Figure S2. Fitting of concentration dependencies of conductance changes (symbols) by Langmuir adsorption isotherms (curves): sorbitol (black circles), glucose (red circles), ethylene glycol (green circles), and fructose (blue circles); Figure S3. The influence of various fructose concentrations on the impedance spectra (a) and electrical resistance (b) of gold electrodes coated with PThBA and a thiophenol sublayer; 20 potential cycles were performed during polymerization. Measurement conditions: without argon bubbling; Figure S4. The influence of various fructose concentrations on the impedance spectra (a) and electrical resistance (b) of gold electrodes coated with PThBA and a thiophenol sublayer; 50 potential cycles were performed during polymerization. Measurement conditions: without argon bubbling; Figure S5. Binding constants towards various analytes at pH 7.4, 8.0, and 8.5
