# Supplementary material for: Conductometric Chemosensor for Saccharides Based on Thin Films of Poly(3-Thienylboronic) Acid: Measurements of Transversal Resistance
Source: Biosensors (Basel). 2025 Oct 9;15(10):679. doi: 10.3390/bios15100679 (PMC12564612; doi:10.3390/bios15100679)
Supplement: Supplementary file 1 [file biosensors-15-00679-s001.zip › biosensors-3820416-supplementary.pdf]

Conductometric Chemosensor for Saccharides Based on Thin Films of Poly(3-Thienylboronic) Acid: Measurements of Transversal Resistance

Berfinsu Kaya, Yulia Efremenko and Vladimir M. Mirsky*

Department of Nanobiotechnology, Institute of Biotechnology, Brandenburg University of Technology Cottbus-Senftenberg, Germany.

*Corresponding author. Tel.: +49 3573 85917; E-mail: mirsky@b-tu.de (V.M. Mirsky)

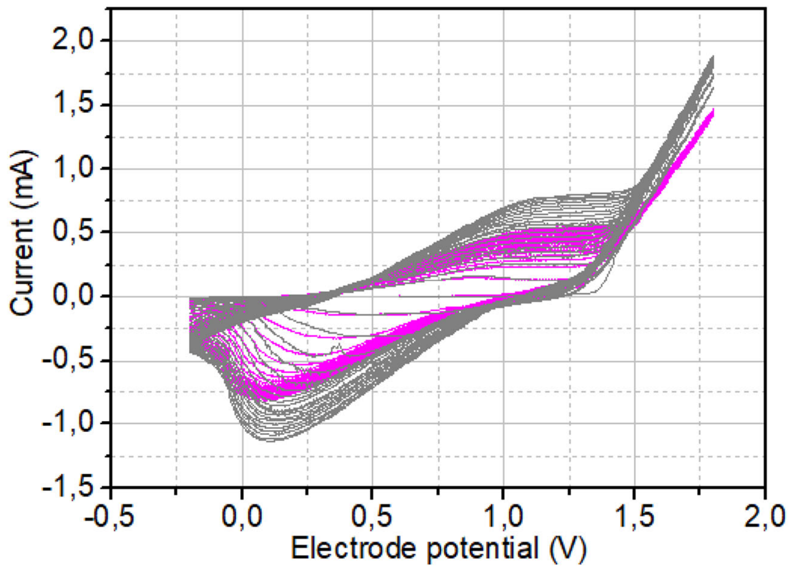

Figure S1. Cyclic voltammetry of electrochemical polymerization of PThBA on the bare gold electrode (red) and on the thiophenol-coated gold electrode (black). Deposition was performed over 20 consecutive cycles, within a potential window of -0.2 V to +1.8 V (vs. Ag/AgCl) at the scan rate of 0.1 V/s. Electrolyte: 50 mM ThBA dissolved in the mixture of 90% BFEE and 10% ACN (v/v).

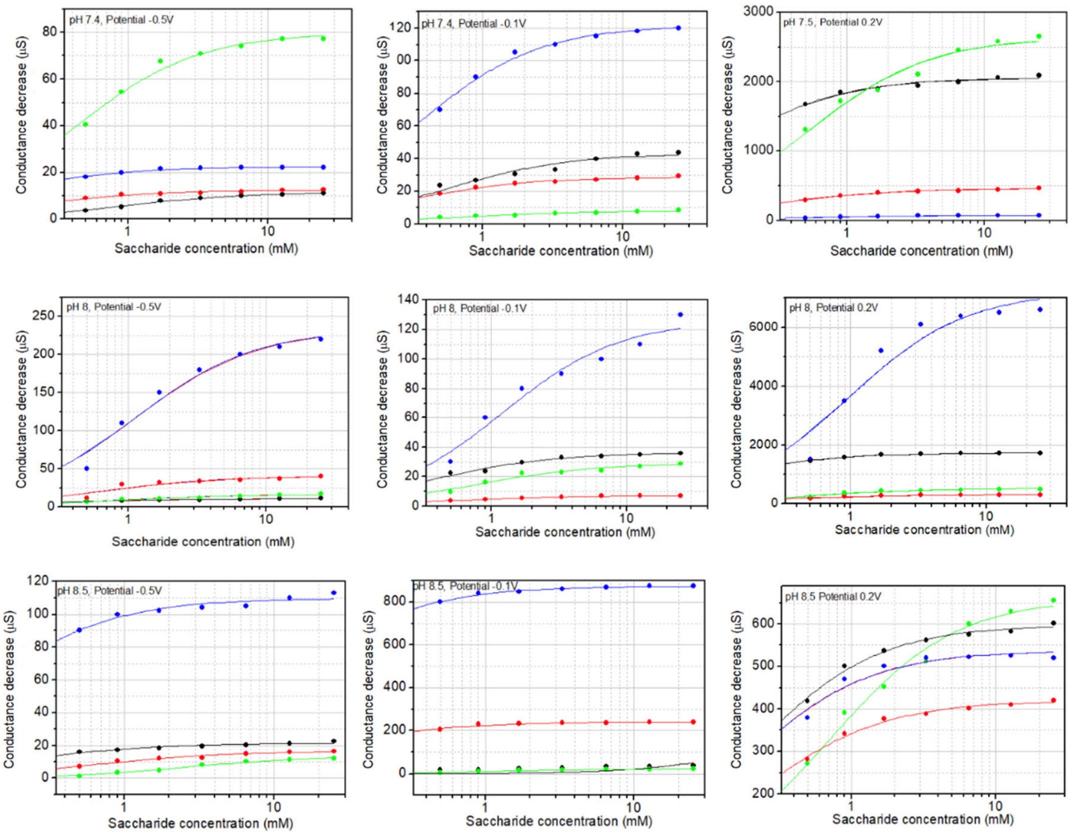

Figure S2. Fitting of concentration dependencies of conductance changes (symbols) by Langmuir adsorption isotherms (curves): sorbitol (black circles), glucose (red circles), ethylene glycol (green circles), and fructose (blue circles).

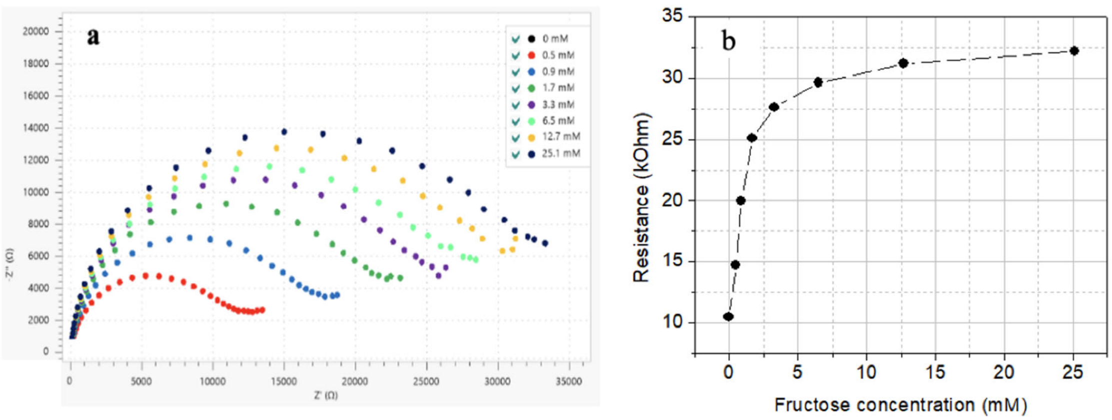

Figure S3. The influence of various fructose concentrations on the impedance spectra (a) and electrical resistance (b) of gold electrodes coated with PThBA and a thiophenol sublayer; 20 potential cycles were performed during polymerization. Measurement conditions: without argon bubbling.

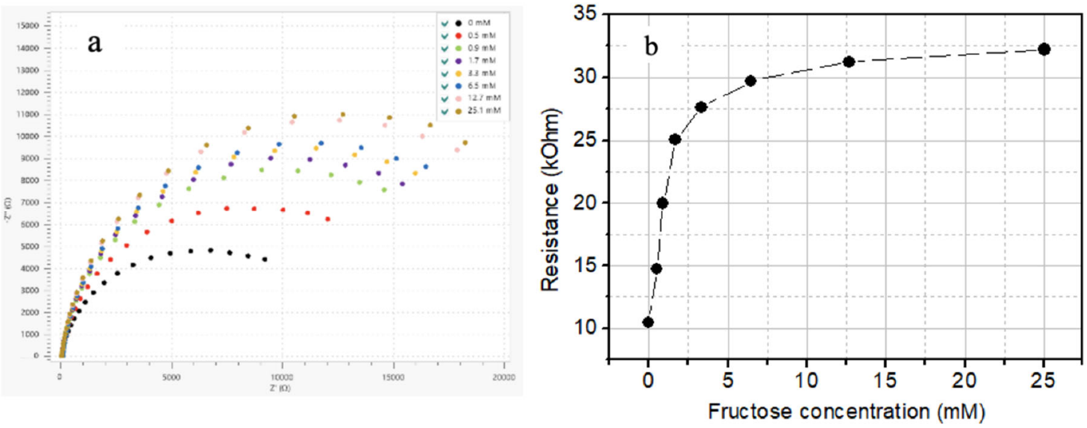

Figure S4. The influence of various fructose concentrations on the impedance spectra (a) and electrical resistance (b) of gold electrodes coated with PThBA and a thiophenol sublayer; 50 potential cycles were performed during polymerization. Measurement conditions: without argon bubbling.

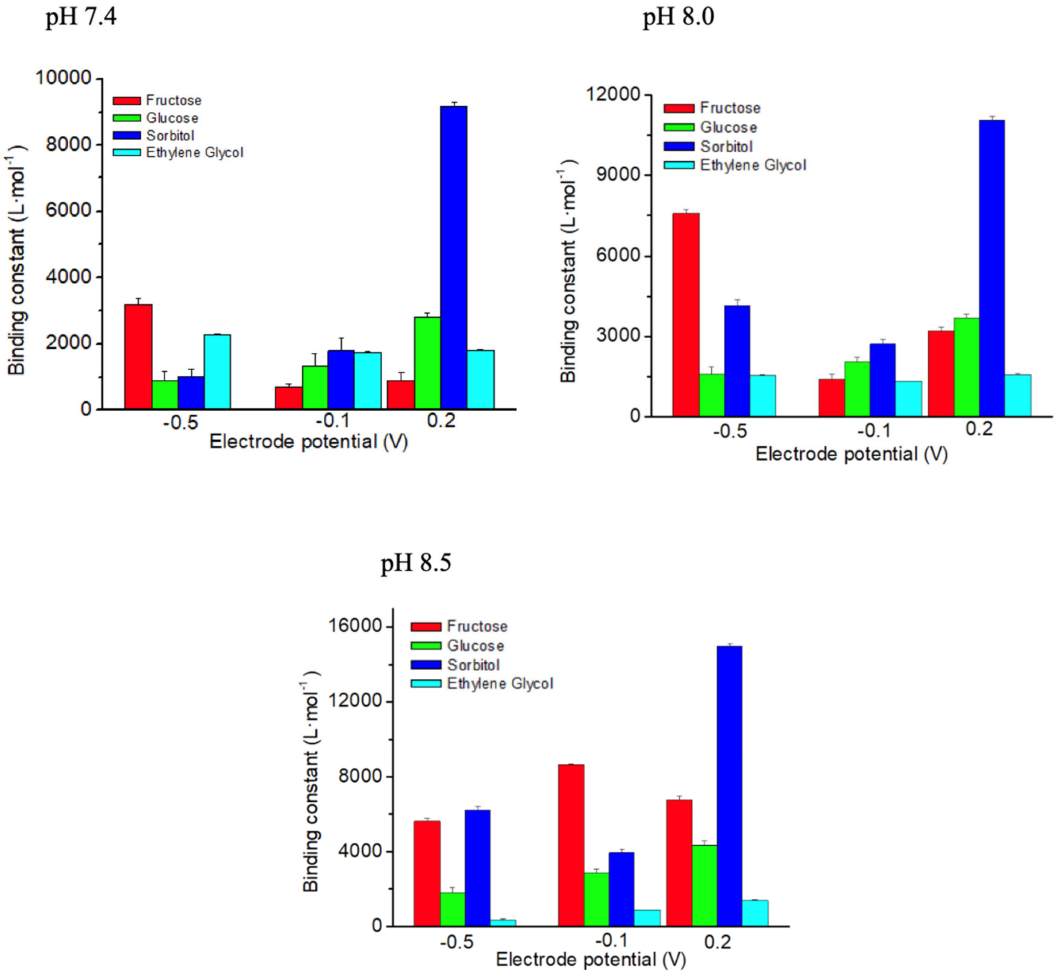

Figure S5. Binding constants towards various analytes at pH 7.4, 8.0, and 8.5.
